# Robot-assisted versus conventional laparoscopic radical hysterectomy in cervical cancer stage IB1

**DOI:** 10.7150/ijms.79830

**Published:** 2023-01-22

**Authors:** Sang Il Kim, Ji Geun Yoo, Sung Jong Lee, Dong Choon Park, Joo Hee Yoon

**Affiliations:** 1 Department of Obstetrics and Gynecology, St. Vincent's Hospital, College of Medicine, The Catholic University of Korea, Seoul, Republic of Korea; 2 Department of Obstetrics and Gynecology, Daejeon St. Mary's Hospital, College of Medicine, The Catholic University of Korea, Seoul, Republic of Korea; 3 Department of Obstetrics and Gynecology, Seoul St. Mary's Hospital, College of Medicine, The Catholic University of Korea, Seoul, Republic of Korea

**Keywords:** cervical cancer, robot surgery, robot-assisted laparoscopic radical hysterectomy, RACC

## Abstract

**Objective:** The aim of this study was to compare survival outcomes of robot-assisted laparoscopic radical hysterectomy (RRH) and conventional laparoscopic radical hysterectomy (LRH) in cervical cancer stage IB1.

**Method:** This is a retrospective study of patients with cervical cancer stage IB1 who surgically treated by either RRH or LRH. Oncologic outcomes of the patients were compared according to surgical approach.

**Results:** In total, 66 and 29 patients were assigned to LRH and RRH groups. All patients had stage IB1 disease (FIGO 2018). Intermediate risk factors (tumor size, LVSI, and deep stromal invasion), proportion of patients receiving adjuvant therapy (30.3% vs. 13.8%, p = 0.09), and median follow-up time (LRH, 61 months; RRH, 50 months; p=0.085) did not differ significantly between the two groups. The recurrence rate was higher in the LRH group; however, there was no significant difference between the two groups (p=0.250). DFS (55.4 vs 48.2 months, p = 0.250), and OS (61.2 vs 50.0 months, p = 0.287) were similar between the LRH and RRH groups.

**Conclusion:** In patients with a tumor size < 2 cm, the recurrence rate was lower in RRH group; however, there was no significant difference. Further large-scale RCTs and clinical studies are required to provide relevant data.

## Introduction

Cervical cancer is one of the most common types of cancer in developing countries 1. In Korea, it is the second most common gynecological cancer. Although the incidence of cervical cancer has been decreasing, it is expected to account for 2,971 new cases and 749 deaths by 2022 2.

The treatment options for newly diagnosed cervical cancer are well established. According to the National Comprehensive Cancer Network (NCCN) guidelines, patients with stage IA, IB, and IIA cancer without bulky masses should be treated surgically 3.

Minimally invasive surgery (MIS) for cervical cancer was first described in 1992 by Nezhat et al. and Canis et al. 4, 5. MIS is associated with lower operative morbidity and fewer postoperative complications than open surgery 6 with similar outcomes 7, 8, leading to an increase in the use of MIS 9. However, in November 2018, the Laparoscopic Approach to Cervical Cancer (LACC) trial reported that MIS was inferior to open surgery, with a hazard ratio (HR) of 3.7 (95% CI 1.63 to 8.58) for recurrence and 6.0 (95% CI 1.77 to 20.3) for overall survival (OS) 10. These results have led to a paradigm shift in the management of cervical cancer, and NCCN and European Society of Gynaecological Oncology (ESGO) have changed the treatment guidelines for early stage cervical cancer 3, 11.

There are several controversies surrounding the LACC trial 12, 13. First, the results may be due to the surgical technique or inexperience of the operator, not because of the MIS itself. The use of a uterine manipulator and intracorporeal colpotomy under CO_2_ pneumoperitoneum may account for the breakdown and spillage of the tumor, and cause tumor dissemination and peritoneal seeding. Second, only 16% of the study participants underwent robot-assisted laparoscopic surgery.

Numerous studies reported comparable outcomes between robot-assisted laparoscopic radical hysterectomy (RRH) and open radical hysterectomy 14 - 16. However, only a few studies compared oncologic outcomes between RRH and conventional laparoscopic radical hysterectomy (LRH); most studies have mainly focused on safety and feasibility 17 - 19.

To prevent intraoperative tumor spillage, *Kanao et al.* introduced the “no-look, no-touch” technique 20. The no-look, no-touch technique includes the following measures: creation of a vaginal cuff, manipulation of the uterus without insertion of a uterine manipulator, minimal handling of the uterine cervix, and bagging of the specimen. Laparoscopic radical hysterectomy using the no-look, no-touch technique showed similar oncologic outcomes to open radical hysterectomy.

With this background, we hypothesized that robot-assisted laparoscopic surgery is superior to conventional laparoscopy when a specific procedure to prevent intraoperative tumor spillage is incorporated. Thus, we decided to evaluate the data from our institution to compare oncologic outcomes in a cohort of women undergoing LRH versus RRH for early stage cervical cancer.

## Materials and Methods

This retrospective cohort study was approved by the Institutional Review Board of the Catholic University of Korea. The requirement for informed consent was waived for this study because of its retrospective nature.

### Study population

From our institution's cancer registry, we identified patients who underwent MIS for cervical cancer between January 2010 and December 2020 at the St. Vincent Hospital. Using the 2018 FIGO staging system, 119 patients who received primary surgical treatment and had histologically confirmed stage IB1 and IB2 disease were initially included. All patients underwent type C radical hysterectomy according to the Querleu-Morrow classification 21. We excluded patients with any of the following characteristics from our analysis: any histologic type other than squamous cell carcinoma, adenocarcinoma, or adenosquamous carcinoma; radiation therapy or neoadjuvant chemotherapy prior to surgery; underwent fertility-sparing surgery or vaginal radical hysterectomy; and insufficient clinical and/or pathologic data. We divided patients who met the study inclusion and exclusion criteria into two groups: those who underwent LRH and those who underwent RRH.

After surgery, adjuvant radiotherapy was selectively implemented according to the Sedlis criteria 22.

In the RRH group, all patients underwent the no-look, no-touch technique to avoid tumor spillage. In the LRH group, the uterine manipulator was used during surgery on a case-by-case basis.

The majority (79.8%) of the study population was stage IB1. All patients with stage IB2 underwent LRH. None of the patients with stage IB2 had undergone RRH. Thus, to minimize the heterogeneity between the two groups, patients with stage IB2 were excluded, and only those with stage IB1 were analyzed.

### Data collection and definitions

We collected information about clinicopathological characteristics (age, histologic type, grade, FIGO stage, tumor size, and risk factors) and adjuvant treatments. The tumor size was documented as the longest diameter based on histopathological findings. Disease-free survival (DFS) was defined as the duration from the date of initial diagnosis to the date of recurrence based on imaging findings, tissue biopsy, or the date of the last follow-up. Overall survival (OS) was defined as the duration from the date of initial diagnosis to the date of cancer-related death or the last follow-up.

### Statistical analysis

The differences in clinicopathological characteristics between the two groups were evaluated using Student's t-test, chi-square test, or Fisher's exact test. We used the Kaplan-Meier method with log-rank tests to compare DFS and OS between the two groups. All statistical analyses were performed using the SPSS statistical software (version 21.0; SPSS Inc., Chicago, IL, USA). Statistical significance was set at P <0.05.

## Results

A total of 95 patients were included in the final analysis. Of these, 66 patients underwent LRH (69.5%), and 29 underwent RRH (30.5%). The clinicopathologic characteristics of the patients are presented in Table 1. The mean age of the patients did not differ between the two groups (52 years in the LRH group and 50 years in the MIS group). Neither group showed a significant difference in histological subtype or grade. All patients had stage IB1 disease. Intermediate risk factors (tumor size, LVSI, and deep stromal invasion), proportion of patients receiving adjuvant therapy (30.3% vs. 13.8%, p = 0.09), and median follow-up time (LRH, 61 months; RRH, 50 months; p=0.085) did not differ significantly between the two groups.

**Figure 1:**
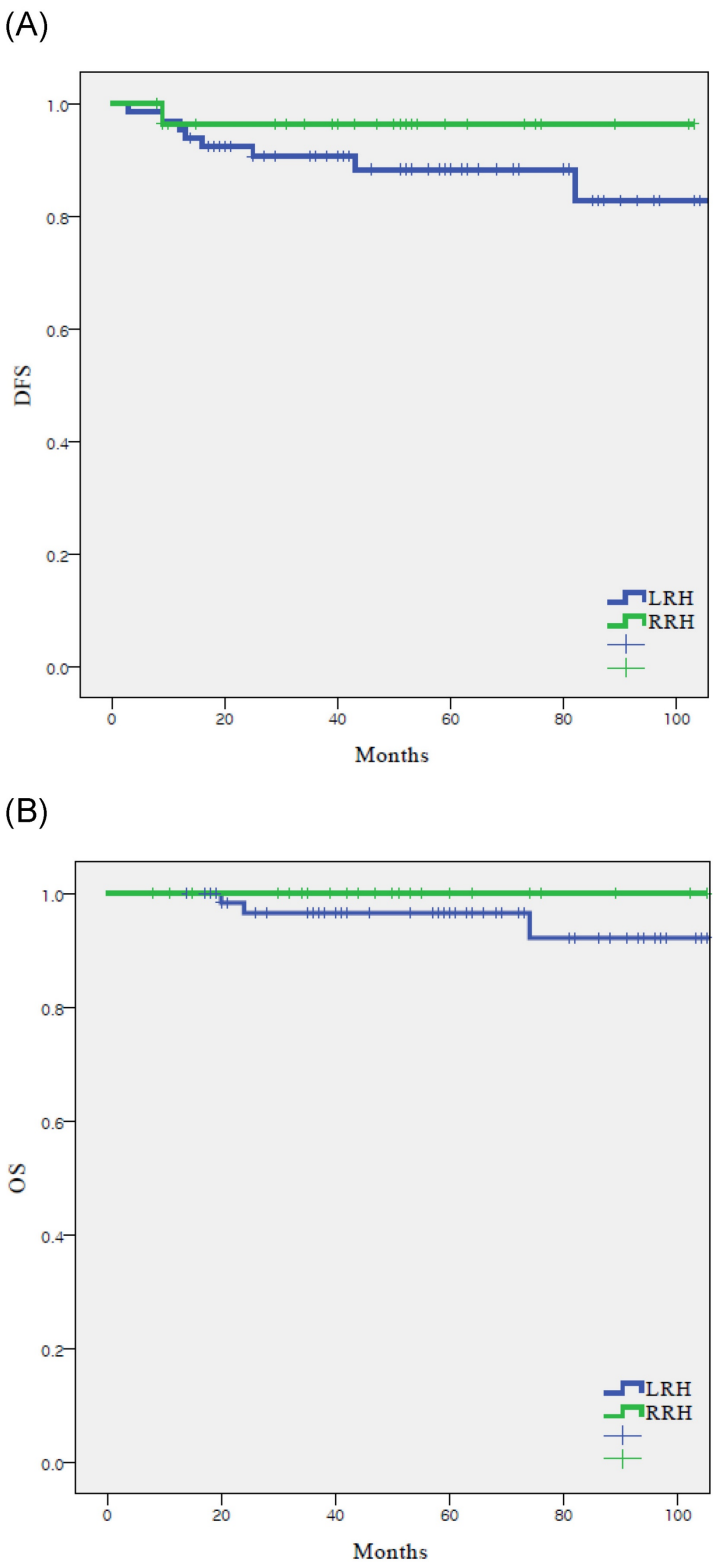
Survival outcomes in study population. All patients. (n = 95) (A) disease-free survival, (B) overall survival

There were nine recurrences (9.5%) in the cohort at the time of analysis (Table 2), eight (12.1%) with LRH and one (3.4%) with RRH. The recurrence rate was higher in the LRH group; however, there was no significant difference between the two groups (p=0.250). In the RRH group, the one case of recurrence occurred in the peritoneum. In the LRH group, five (62.5%) of the eight recurrences were locoregional. There were three (3.2%) cancer-related deaths in the entire cohort, which all occurred in the LRH group (4.5%). DFS (55.4 vs 48.2 months, p = 0.250), and OS (61.2 vs 50.0 months, p = 0.287) were similar between the LRH and RRH groups (Fig. 1).

The Cox proportional hazards model was used to evaluate the prognostic factors for recurrence (Table 3). Univariate analysis revealed that none of the factors were significantly associated with DFS.

## Discussion

The use of MIS in gynecologic oncology was first reported in 1992 23, and numerous studies have compared MIS with open surgery in cervical cancer 6, 9, 24. MIS has been accepted as an alternative to open surgery with reduced operative morbidity and postoperative complications, and comparable outcomes. In addition to conventional laparoscopy, robot-assisted laparoscopic surgery was approved by the Food and Drug Agency for gynecological indications in 2005. Since then, the use of robotic surgery in gynecologic oncology has increased. The safety and effectiveness of robotic surgery in cervical cancer has been reported several times 25, 26.

The results of the LACC trial contradicted those of previous studies and questioned the safety of MIS 10, leading to changes in treatment guidelines and protocols; NCCN and ESGO guidelines no longer recommend MIS in cervical cancer 3, 11. One criticism of the results of the LACC trial is the low rate of RRH (16%). In contrast, in 2018, more than 80% of radical hysterectomies were performed using robot-assisted laparoscopic surgery in Sweden 27. Therefore, the LACC trial may not reflect current practice, especially in countries that have adopted robotic platforms for gynecologic oncology. After the results of the LACC trial were reported, many retrospective studies, including those rebutting or following the LACC trial, have been reported 27 - 31. Some studies reported comparable oncologic outcomes between RRH and open radical hysterectomy 32 - 34. However, no studies have shown the superiority of RRH compared with LRH.

In this hospital-based retrospective analysis, we compared the oncological outcomes of RRH and LRH for the treatment of cervical cancer stage IB1. In our cohort, we observed no differences in the clinicopathological characteristics between the two groups. The rate of adjuvant treatment was higher in the LRH group, but the difference was not statistically significant.

In our cohort, the recurrence rates were 12.1% and 3.4% in the LRH and RRH groups, respectively. The recurrence rate in the LRH group of our study was similar to that in the MIS group in the LACC trial (8.5%), and the recurrence rate in the RRH group of our study was similar to that in the open group in the LACC trial (2.2%).

The lower recurrence rate in the RRH group can be explained by several factors. The first factor is the considerable benefits of the robotic system. The robotic system provides improved three-dimensional vision, a more ergonomic surgeon position, and articulated wrist-like instruments, thereby increasing surgical precision and dexterity 35. As LRH is generally known to be one of the most difficult surgeries in the field of gynecologic oncology, the benefits of the robotic system might cause differences in oncologic outcomes. Second, the no-look, no-touch technique was used to prevent intraoperative tumor spillage and dissemination.

Although the recurrence rate was not significantly different between the two groups, our results indicate that RRH had favorable outcomes compared with LRH in patients with stage IB1. All patients in our study had a tumor size of < 2 cm, and RRH for tumors less than 2 cm appeared to be safer. These results are in concordance with a previous study by *Doo et al.* that analyzed survival outcomes in patients with stage IB1 tumors; patients with tumors ≥ 2 cm undergoing RRH had a shorter DFS 34. Our results suggest that in patients with a tumor size < 2 cm, RRH must be considered if the surgeon is using MIS as a surgical approach.

Our study had several limitations. First, due to the retrospective study design, there may have been inevitable issues such as selection bias. Second, the sample size and observation period may have been insufficient to properly compare oncologic outcomes between the two groups. Third, perioperative morbidity was not evaluated. Forth, variations in techniques, expertise, and outcomes among surgeons were not considered.

In conclusion, in patients with a tumor size < 2 cm, the recurrence rate was lower in RRH group; however, there was no significant difference. Further large-scale RCTs and clinical studies are required to provide relevant data. We expect to obtain the results of ongoing large-scale RCTs (NCT03719547, NCT04831580).

## Figures and Tables

**Table 1:** Clinopathological characteristics of patients according to surgical approach (n = 95)

	LRH (n = 66, %)	RRH (n = 29, %)	P value
Age, years Mean ± SD	52.21 ± 1.416	50.66 ± 1.568	0.766
Histologic type SCC ACC ASC	50 (75.8) 12 (18.1) 4 (6.1)	21 (72.4) 8 (27.6) 0 (0)	0.822
Grade 1 2 3	19 (28.8) 45 (68.2) 2 (3.0)	11 (37.9) 18 (62.1) 0 (0)	0.277
Tumor size (cm) Mean ± SD	1.235 ± 0.615	1.100 ± 0.788	0.062
LVSI (+)	12 (18.2)	5 (17.2)	0.913
Deep stromal invasion	7 (10.6)	1 (3.4)	0.250
Adjuvant RT	20 (30.3)	4 (13.8)	0.090
Follow-up time (months) Median, range	61 14 - 122	50 8 - 105	0.085

LRH, conventional laparoscopic radical hysterectomy; RRH, robot-assisted laparoscopic radical hysterectomy; SD, standard deviation; SCC, squamous cell carcinoma; ACC, adenocarcinoma; ASC, adenosquamous carcinoma; LVSI, lymphovascular space invasion; RT, radiation therapy

**Table 2:** Oncologic survival outcomes according to surgical approach (n = 95)

	LRH (n = 66, %)	RRH (n = 29, %)	P value
Recurrence	8 (12.1)	1 (3.4)	0.250
Site of recurrence, total Stump Pelvic lymph node Lung Peritoneum	8 3 (37.5) 2 (25.0) 2 (25.0) 1 (12.5)	1 0001 (100)	0.261
Death	3 (4.5)	0	0.287

LRH, conventional laparoscopic radical hysterectomy; RRH, robot-assisted laparoscopic radical hysterectomy

**Table 3:** Univariate and multivariate analysis of prognostic factors for disease-free survival (n = 95)

Characteristics	Univariate analysis			Multivariate analysis		
	OR	95% CI	*p value*	OR	95% CI	*p value*
Surgical approach LRH RRH	1 (Ref) 0.315	- 0.039 - 2.527	- 0.251			
Histologic type SCC ACC ASC	1 (Ref) 3.233 -	- 0.867 - 12.062 -	- 0.081 0.987			
Grade 1 2 3	1 (Ref) 4.160 -	0.519 - 33.334 -	0.179 0.992			
LVSI Negative Positive	1 (Ref) 0.536	- 0.066 - 4.326	- 0.526			
Deep stromal invasion						
No	1 (Ref)	-	-			
Yes	1.120	0.139 - 9.017	0.915			
Adjuvant RT No Yes	1 (Ref) 0.789	- 0.163 - 3.820	- 0.769			

Covariates with *p* < 0.05 on univariate analysis were included in multivariate model.OR, odds ratio; CI, confidence interval; Ref, reference; LRH, conventional laparoscopic radical hysterectomy; RRH, robot-assisted laparoscopic radical hysterectomy; SCC, squamous cell carcinoma; ACC, adenocarcinoma; ASC, adenosquamous carcinoma; LVSI, lymphovascular space invasion; RT, radiation therapy.
